# 江苏省昆山市1981年-2015年肺癌死因对期望寿命和潜在减寿年影响分析

**DOI:** 10.3779/j.issn.1009-3419.2017.09.04

**Published:** 2017-09-20

**Authors:** 文斌 胡, 婷 张, 威 秦, 建国 史, 岚 仝, 和泉 邱, 杰 周, 亦徐 金, 晓明 罗, 月平 沈

**Affiliations:** 1 215300 昆山，江苏省昆山市疾病预防控制中心 慢性非传染性疾病预防控制科 Department of Chronic Non-Communicable Disease Preventive and Control, Kunshan Center for Disease Control and Prevention, Kunshan 215300, China; 2 215300 昆山，昆山高新区社区卫生服务中心 Kunshan High-tech Zone Jiangpu Community Health Services Center, Kunshan, 215300, China; 3 215123 苏州，苏州大学医学部公共卫生学院流行病与卫生统计学系 Department of Epidemiology and Medical Statistics, School of Public Health, Medical College of Soochow University, Suzhou 215123, China

**Keywords:** 肺肿瘤, 潜在减寿年, 年度变化百分比, 去肿瘤死因期望寿命, Lung neoplasms, Potential years of life lost, Annual percentage change, Cancer eliminated life expectancy

## Abstract

**背景与目的:**

肺癌发病率和死亡率在男性和女性人群持续上升，但是有关肺癌所致的潜在减寿年和去肺癌死因可增加期望寿命时间变化趋势的研究较少。本研究旨在探究江苏省昆山市1981年-2015年去肺癌死因可增加期望寿命和肺癌所致的潜在减寿年时间趋势。

**方法:**

1981年-2015年肺癌死亡病例来源于死因监测，以计算去肺癌死因可增加期望寿命和潜在减寿年。用中国2000年第五次人口普查的年龄结构计算分性别的年龄标化潜在减寿年。使用年度变化百分比（estimate annual percentage change, eAPC）评价去肺癌可增加期望寿命和潜在减寿年在年份之间变化趋势。

**结果:**

总人群中去肺癌死因可增加期望寿命由1981年的0.34岁上升到2015年的0.86岁，上升趋势有统计学意义（APC=3.2%, 95%CI: 2.8%-3.6%）；男性人群（APC=3.0%, 95%CI: 2.5%-3.5%）和女性人群中（APC=3.6%, 95%CI: 3.0%-4.2%）去肺癌死因可增加期望寿命也呈现上升趋势。肺癌所致的标化潜在减寿年在总人群（APC=-0.1%, 95%CI: -0.6%-0.4%）和男性人群（APC=-0.5%, 95%CI: -1.1%~0.1%）无明显趋势变化，而女性人群中明显上升（APC=1.5%, 95%CI: 0.3%-2.7%）。

**结论:**

虽然因肺癌所致的过早死亡没有趋势变化，而肺癌死因对全人群期望寿命的影响在持续上升，有针对性的肺癌预防控制措施亟需开展。

## Abstract (en)

**Background and objective:**

Previous reports have shown that lung cancer incidence and mortality were increasing both in male and female; however, the temporal trends in lung cancer eliminated life expectancy and potential years of life lost (PYLL) are very rare. Thus, we examine the temporal trends in lung cancer eliminated life expectancy and PYLL in Kunshan city, Jiangsu province, 1981-2015.

**Methods:**

Data were collected from vital registry of Kunshan city. Lung cancer eliminated life expectancy and the PYLL were calculated by sex. The Chinese population in 2000 was used to calculate age-standardized PYLL. Estimate annual percentage changes (eAPC) and 95% confidence interval (CI) were used to examine the temporal trendss.

**Results:**

During 1981 to 2015, substantially increasing trend was observed for the lung cancer eliminated life expectancy, which increased by 0.34 years in 1981 to 0.86 in 2015 (APC=3.2%, 95%CI: 2.8%-3.6%), and a significant increasing trend was found for male (APC=3.0%, 95%CI: 2.5%-3.5%) and female (APC=3.6%, 95%CI: 3.0%-4.2%). Moreover, the age-standardized PYLL among both sex (APC=-0.1%, 95%CI: -0.6%-0.4%) and male (APC=-0.5%, 95%CI: -1.1%-0.1%) were stable, but increasing trend was observed in females (APC=1.5%, 95%CI: 0.3%-2.7%).

**Conclusion:**

Although there was no significant change over the past 3 decades regarding the effect of premature deaths due to lung cancer, a substantial increasing trend was observed in lung cancer eliminated life expectancy, which suggested that targeted lung cancer prevention and control measures are urgently need.

恶性肿瘤是中国第2大死因，肺癌不但在恶性肿瘤死因中位居第一^[1]^，而且当前肺癌发病率和死亡率均呈现明显上升趋势^[2]^，但是长期连续呈现肺癌疾病负担的研究较为少见。因此，本研究基于覆盖户籍人群全死因监测，在掌握全死因死亡率^[3]^和平均期望寿命变化趋势的基础上^[4]^，跟踪分析昆山市连续35年（1981年-2015年）去肺癌死因期望寿命与潜在减寿年，以期为昆山地区肺癌预防与控制提供参考，也为将来评价肺癌相关的防治效果提供依据。

## 1 对象与方法

### 1.1 数据来源

江苏省昆山市于1981年全面开展覆盖全户籍人口的全死因监测工作^[3, 4]^。年初与年末人口数来源于昆山市公安局，以计算年均观察人年数，1981年-2014年年均观察人年数（年均人口数）详见文献^[4]^。2015年年均人年数在总人群、男性和女性分别为778, 389人年、386, 281人年和392, 108人年。平均期望寿命以及去肺癌死因期望寿命计算采用蒋庆琅法^[5]^。去肺癌可增加期望寿命=去肺癌死因期望寿命-平均期望寿命。

潜在减寿年（potential years of life lost, PYLL）是指某病某年龄组人群死亡的期望寿命与实际死亡年龄之差的总和，即死亡造成的寿命损失。计算公式如下^[6]^：

$
			{\rm{PYLL = }}\sum \left[{\left( {{\rm{E-X}}} \right) \times  = {{\rm{D}}_{\rm{i}}}} \right]
			$

其中E为期望寿命（本研究定为70岁，主要看1岁-69岁因肺癌死亡所造成的过早死亡），X为各个年龄组的组中值，Di观察人群各个年龄组的死亡人数。

标化潜在减寿年为：

$
			{\rm{SPYLL \left( {{\rm{人年}}} \right)= }}\sum \left( {\frac{{{\rm{年龄组PYLL}}}}{{{\rm{年龄组人数}}}} \times {\rm{校正因子}}} \right) \times 1000
			$

其中，校正因子=标准人口年龄别构成/观察人群年龄构成。

标化潜在减寿率= SPYLL/N*1, 000，其中N为1岁-69岁人群人口数。

### 1.2 统计学分析

采用SAS 9.3软件，基于历年分年龄组的年均人口数，计算分年份、性别和年龄组的平均期望寿命、PYLL、标化的PYLL以及潜在减寿率，年龄域采用1岁-69岁，按5岁一个年龄组。按照第五次全国人口普查（2000年）人口年龄结构为标准计算年龄标化潜在减寿年。采用对数线性回归法计算年度变化百分比（annual percent change, APC）及其95%CI来评价期望寿命和潜在减寿率等指标的时间趋势，以APC其95% CI是否包含0为统计学显著性检验标准^[7]^。

## 2 结果

### 2.1 江苏省昆山市1981年-2015年去肺癌期望寿命分析

从肺癌占全死因构成比看，总人群中肺癌占全死因构成比由1981年的2.8%上升到2015年的8.2%，上升趋势有统计学意义（APC=3.7%, 95%CI: 3.4%-4.0%）；男性人群（APC=3.6%, 95%CI: 3.3%-3.6%）和女性人群中（APC=4.2%, 95%CI: 3.6%-4.8%）肺癌占全死因构成比也呈现上升趋势，见表 1。

**1:** 江苏省昆山市1981年-2015年去肺癌死因期望寿命分析 Lung cancer eliminated life expectancy in Kunshan city, Jiangsu province, 1981-2015

Years	Proportion of all cause death (%)				Proportion of all cancer death (%)				Increased by lung cancer eliminated life expectancy (years)				Proportion of lung cancer to all cancer eliminated life expectancy (%)		
	Both	Male	Female		Both	Male	Female		Both	Male	Female		Both	Male	Female
1981	2.8	4.5	1.0		12.0	16.0	5.1		0.34	0.51	0.11		10.6	14.1	4.3
1982	3.0	4.2	1.4		11.8	14.5	7.0		0.35	0.45	0.18		10.4	12.3	6.5
1983	2.8	3.7	1.6		12.1	13.8	9.0		0.32	0.68	0.21		11.0	21.1	9.1
1984	2.9	4.1	1.5		11.4	13.9	7.4		0.36	0.47	0.20		10.3	12.2	6.8
1985	3.4	4.6	2.0		13.3	16.0	9.3		0.40	0.53	0.23		11.6	13.9	7.8
1986	3.0	4.6	1.0		12.4	16.3	5.3		0.37	0.55	0.12		11.0	14.2	4.6
1987	3.1	4.4	1.6		13.0	15.6	8.5		0.36	0.48	0.20		11.1	13.0	6.1
1988	3.3	4.9	1.5		13.7	16.8	8.2		0.40	0.53	0.19		12.3	14.6	7.5
1989	2.9	4.1	1.3		11.6	14.7	6.5		0.33	0.43	0.16		10.4	12.7	5.9
1990	3.3	4.9	1.3		11.9	14.5	6.5		0.35	0.48	0.14		10.2	12.0	5.6
1991	4.2	5.8	2.3		15.6	18.9	10.2		0.48	0.60	0.26		14.1	16.4	9.2
1992	3.8	5.7	1.8		15.0	18.4	9.2		0.45	0.62	0.24		13.3	15.7	9.0
1993	3.7	5.6	1.5		14.5	18.0	7.7		0.41	0.59	0.16		12.8	15.4	6.7
1994	4.2	5.8	2.2		16.9	19.8	11.3		0.45	0.61	0.23		15.6	17.6	10.8
1995	4.3	6.1	2.0		16.6	19.2	10.6		0.47	0.64	0.21		14.7	16.2	9.7
1996	4.3	6.1	2.2		15.5	18.6	10.1		0.47	0.62	0.25		13.4	15.6	9.1
1997	5.0	7.1	2.6		18.1	21.8	11.7		0.58	0.89	0.30		15.9	19.6	10.7
1998	5.0	7.0	2.7		17.6	20.6	12.2		0.56	0.80	0.30		15.6	17.5	11.7
1999	5.9	8.4	2.8		19.2	22.2	12.8		0.65	0.95	0.30		17.0	18.9	11.6
2000	5.4	8.1	2.1		18.5	23.6	9.4		0.59	0.93	0.25		16.6	20.9	9.4
2001	6.1	8.2	3.5		20.0	22.3	15.4		0.66	0.92	0.37		17.7	19.4	13.6
2002	6.4	8.6	3.8		21.2	24.2	16.0		0.78	1.09	0.45		18.8	20.1	14.8
2003	6.1	8.5	3.2		20.6	23.7	14.5		0.65	0.92	0.34		18.1	21.1	12.8
2004	6.8	9.7	3.3		21.0	26.1	12.3		0.76	1.04	0.36		18.6	22.4	11.1
2005	6.8	9.3	3.5		22.0	26.6	13.6		0.72	0.97	0.37		19.0	22.2	12.5
2006	7.5	10.0	4.3		22.0	26.4	14.9		0.80	1.11	0.44		19.3	22.7	13.2
2007	7.8	10.5	4.4		22.8	27.2	15.4		0.82	1.16	0.44		20.4	23.6	14.1
2008	6.9	9.8	3.3		21.8	26.1	13.3		0.68	0.91	0.33		19.2	22.3	12.3
2009	6.3	8.5	3.8		19.1	22.0	14.3		0.62	0.77	0.40		16.8	18.3	13.6
2010	7.5	10.4	3.9		23.5	27.7	15.7		0.75	0.98	0.39		20.8	23.4	15.3
2011	7.5	10.5	4.1		22.8	27.5	15.0		0.79	1.05	0.43		21.2	24.8	14.4
2012	8.4	12.0	4.1		27.1	33.0	16.6		0.83	1.13	0.43		24.1	28.7	16.0
2013	7.7	10.8	4.3		25.0	28.2	18.8		0.77	1.04	0.44		22.4	24.3	17.9
2014	9.0	13.1	4.2		26.9	33.0	16.2		0.91	1.29	0.43		23.9	28.0	14.9
2015	8.2	11.9	4.0		24.9	29.9	15.9		0.86	1.21	0.42		22.3	26.1	14.6
APC (%)	3.7	3.6	4.2		2.6	2.4	3.1		3.2	3.0	3.6		2.6	2.2	3.3
95%CI	3.4-4.0	3.3-3.9	3.6-4.8		2.4-2.8	2.1-2.7	2.6-3.6		2.8-3.6	2.5-3.5	3.0-4.2		2.3-2.9	1.8-2.6	2.7-3.9
APC: annual percentage change.															

从肺癌死因占全肿瘤死因构成比看，肺癌占全肿瘤死因构成比由1981年的12.0%上升到2015年的24.9%，上升趋势有统计学意义（APC=2.6%, 95%CI: 2.4%-2.8%）；男性人群（APC=2.4%, 95%CI: 2.1%-2.7%）和女性人群中（APC=3.1%, 95%CI: 2.6%-3.6%）肺癌占全肿瘤死因构成比也呈现上升趋势，见表 1。

总人群去肺癌死因可增加期望寿命由1981年的0.34岁上升到2015年的0.86岁，上升趋势有统计学意义（APC=3.2%, 95%CI: 2.8%-3.6%）；男性人群去肺癌死因可增加期望寿命（APC=3.0%, 95%CI: 2.5%-3.5%）和女性人群去肺癌死因可增加期望寿命（APC=3.6%, 95%CI: 3.0%-4.2%）也呈现上升趋势。

从去肺癌死因占去肿瘤死因期望寿命的比例看，总人群去肺癌死因占去肿瘤死因比例由1981年的10.6%上升到2015年的22.3%，上升趋势有统计学意义（APC=2.6%, 95%CI: 2.3%-2.9%）；男性人群（APC=2.2%, 95%CI: 1.8%-2.6%）和女性人群（APC=3.3%, 95%CI: 2.8%-3.9%）去肺癌死因占去肿瘤死因期望寿命比例也呈现上升趋势，见表 1。

### 2.2 江苏省昆山市1981年-2015年肺癌所致的潜在减寿年分析

表 2显示因肺癌所致潜在减寿年在总人群、男性人群及女性人群呈现下降趋势。因肺癌所致的标化潜在减寿年在总人群（APC=-0.1%, 95%CI: -0.6%-0.4%）和男性人群（APC=-0.5%, 95%CI: -1.1%-0.1%）无明显趋势变化，而女性人群中肺癌所致的标化潜在减寿年呈现上升趋势（APC=1.5%, 95%CI: 0.3%-2.7%）。

**2:** 江苏省昆山市1981年-2015年因肺癌所致潜在寿命损失 Potential years of life lost (PYLL) of lung cancer in Kunshan city, Jiangsu province, 1981-2015

Years	PYLL (person years)				SPYLL (person years)				Rate of SPYLL (‰)		
	Both	Male	Female		Both	Male	Female		Both	Male	Female
1981	685.0	625.0	60.0		750.8	687.5	66.7		1.5	2.7	0.3
1982	770.0	612.5	157.5		825.6	658.3	171.0		1.6	2.6	0.7
1983	872.5	540.0	332.5		986.7	600.9	389.3		1.9	2.3	1.5
1984	870.0	655.0	215.0		910.5	660.9	252.2		1.8	2.5	1.0
1985	1, 047.5	870.0	177.5		1, 066.0	879.4	177.6		2.1	3.4	0.7
1986	837.5	752.5	85.0		802.7	719.0	77.9		1.6	2.8	0.3
1987	735.0	480.0	255.0		696.3	442.4	258.0		1.3	1.7	1.0
1988	982.5	797.5	185.0		940.1	749.2	182.8		1.8	2.8	0.7
1989	772.5	615.0	157.5		713.8	557.5	153.2		1.4	2.1	0.6
1990	780.0	610.0	170.0		685.2	531.2	159.0		1.3	2.0	0.6
1991	1, 050.0	785.0	265.0		898.4	646.7	256.0		1.7	2.4	1.0
1992	947.5	632.5	315.0		845.0	537.6	310.3		1.6	2.0	1.2
1993	990.0	827.5	162.5		806.2	673.0	119.6		1.5	2.5	0.4
1994	1, 205.0	940.0	265.0		1, 035.2	797.8	234.5		1.9	2.9	0.9
1995	892.5	685.0	207.5		701.0	526.2	158.2		1.3	1.9	0.6
1996	1, 180.0	842.5	337.5		894.1	621.4	264.7		1.6	2.3	1.0
1997	1, 380.0	1, 000.0	380.0		1, 107.0	776.1	324.3		2.0	2.8	1.2
1998	1, 220.0	825.0	395.0		932.3	610.2	313.1		1.7	2.2	1.2
1999	1, 307.5	955.0	352.5		991.3	688.3	294.2		1.8	2.5	1.1
2000	1, 302.5	927.5	375.0		977.9	658.2	309.0		1.8	2.4	1.1
2001	1, 292.5	880.0	412.5		930.6	622.6	302.1		1.7	2.2	1.1
2002	1, 407.5	985.0	422.5		996.5	687.7	309.1		1.8	2.5	1.1
2003	1, 180.0	757.5	422.5		807.6	510.2	294.3		1.4	1.8	1.1
2004	1, 292.5	1, 032.5	260.0		876.7	704.5	167.4		1.5	2.4	0.6
2005	1, 317.5	890.0	427.5		929.2	626.9	297.9		1.6	2.1	1.0
2006	1, 182.5	735.0	447.5		772.8	472.5	298.3		1.3	1.5	1.0
2007	1, 192.5	925.0	267.5		802.1	609.2	184.1		1.3	2.0	0.6
2008	1, 000.0	715.0	285.0		636.6	437.6	198.7		1.0	1.4	0.6
2009	1, 075.0	680.0	395.0		662.0	397.8	262.8		1.0	1.2	0.8
2010	1, 170.0	862.5	307.5		719.7	510.3	209.0		1.1	1.6	0.7
2011	1, 567.5	1, 012.5	555.0		1, 128.1	733.6	386.9		1.7	2.2	1.2
2012	1, 367.5	962.5	405.0		872.6	612.0	263.8		1.3	1.8	0.8
2013	1, 140.0	742.5	397.5		703.3	466.2	238.0		1.0	1.4	0.7
2014	1, 330.0	1, 037.5	292.5		833.8	648.6	186.4		1.2	1.9	0.5
2015	1, 380.0	1, 002.5	377.5		888.1	636.6	251.1		1.3	1.8	0.7
APC (%)	1.6	1.2	3.3		-0.1	-0.5	1.5		-1.1	-1.4	0.4
95%CI	1.1~2.1	0.7~1.7	2.1~4.5		-0.6~0.4	-1.1~0.1	0.3~2.7		-1.7~-0.5	-2.0~-0.8	-0.9~1.7

总人群中因肺癌所致的标化潜在减寿率由1981年的1.5‰下降到2015年的1.3‰（APC=-1.1%, 95%CI: -1.7%--0.5%）。男性人群（APC=-1.4%, 95%CI: -2.0%--0.8%）中因肺癌所致的标化潜在减寿率明显上升，但女性人群（APC=0.4%, 95%CI: -0.9%-1.7%）肺癌所致的标化潜在减寿率无明显趋势变化。

## 3 讨论

本研究从肺癌对平均期望寿命及潜在减寿年两个角度来评价肺癌对人群健康的影响。结果提示：虽然在过去35年间（1981年-2015年）肺癌对1-69岁人群过早死亡无明显变化，但是肺癌对全人群平均期望寿命的影响在持续上升。

当前肺癌发病率和死亡率在不同地区均明显上升^[8-11]^。昆山市肺癌发病和死亡率上升尤其明显^[12, 13]^，进一步分析发现，人口因素（人口老龄化）对肺癌死亡率贡献率达57.28%^[14]^，并且未来年份肺癌死亡率继续呈现上升趋势^[12]^。目前，恶性肿瘤死亡情况在昆山地区发生了巨大变化，肿瘤死亡率总体上呈现下降趋势，癌症死亡谱由原来（1981年）以消化系统癌症为主向当前肺癌死亡为主的转变^[15]^。在癌症死亡顺位中肺癌位居第1位，在2015年占全部癌症死亡的24.9%，并且这种趋势在1981年-2015年期间呈现显著上升趋势^[15]^。

本研究中对去肺癌死因可增加期望寿命和标化潜在减寿年趋势分析发现，肺癌对男性和女性人群平均期望寿命的影响在持续上升。林艺兰^[16]^对厦门肺癌所致的减寿分析发现，肺癌对潜在减寿年的影响逐年上升，而且未来继续呈现上升趋势。与昆山市地理位置相邻的常熟市，肺癌死亡率在1973年-2010年期间持续上升，且因肺癌所致寿命损失十分巨大^[17]^。本研究提示肺癌所致标化潜在减寿率在男性人群明显下降，而在女性人群无明显趋势变化。从时间趋势看，虽然肺癌对男性人群的危害要比女性大，但是男性人群中肺癌所致的标化潜在减寿年无明显时间趋势，而女性人群中肺癌所致标化潜在减寿年随着时间而上升。这提示肺癌所致的过早死亡在男性人群中高水平维持，而在女性人群中低水平持续上升。

肺癌对人群健康危害受到发病率和死亡率两方面影响。对中国2003年-2005年肿瘤随访发现，中国肺癌相对生存率仅为16.1%，城市远大于农村地区（19.5% *vs* 11.2%），提示社会经济发展对肺癌预后的影响。研究显示人类发展指数与肺癌死亡发病比呈现负相关^[18]^。与此相似的是，陈万青对城市化率与恶性肿瘤流行进行了分析，提示区域性社会经济发展与特定癌症明显关联^[19]^。但是这种差异不足以区分性别之间肺癌疾病负担。研究显示肺癌组织学分类构成发生了较大变化，肺腺癌呈现持续上升趋势，而鳞癌则呈现下降趋势^[20, 21]^。对浦东新区肺癌组织学类型研究中，腺癌占21.1%、鳞癌占13.9%^[22]^。北京城区1988年-2007年期间，男性腺癌和鳞癌各占36.68%和35.13%；女性人群中腺癌和鳞癌各占60.83%和14.77%，可见女性人群中肺腺癌是主要组织学类型，是影响女性健康的主要因素。肺癌发病受到遗传、生活方式、环境因素及等多种因素影响，明确的因素有吸烟与二手烟暴露、空气污染^[23]^、室内空气暴露^[24]^等。但是与女性肺腺癌发生相关的因素尚待进一步研究。

肺癌流行及其变化趋势是一系列因素综合作用的表现，而肺癌对健康影响是发病率、死亡率、早诊早治、医疗卫生水平等因素集中体现。就肺癌早发现，研究显示低剂量计算机断层扫描筛查肺癌可能有利于具有肺癌高风险个体^[25]^。2014年，美国预防服务工作组发布了肺癌筛查推荐，推荐级别为B级，将筛查年龄延长至80岁，这将使整体肺癌死亡率降低14%，符合筛查标准人群的肺癌死亡率减少25%，使获益与损害达到很好的平衡^[26, 27]^。但这仅仅是来源于美国人群的证据，是否适合中国某个特定目标人群，其高危人群定义、筛查范围、筛查路径以及较高的成本效益均需要进一步循证论证。

总之，本研究提示肺癌对全人群过早死亡的影响无明显趋势变化，但是去肺癌死因可增加期望寿命呈现了显著上升趋势。可见，肺癌对全人群健康影响在昆山地区明显持续上升。在人口老龄化背景下切实开展肺癌相关的一级预防及健康促进行动，尽早实施以肺癌早诊为特征的二级预防，是从根本上应对肺癌发生、减小肺癌疾病负担的有效措施。
